# Repurposing the Open Global Health Library for the discovery of novel Mpro destabilizers with scope as broad-spectrum antivirals

**DOI:** 10.3389/fphar.2024.1390705

**Published:** 2024-07-10

**Authors:** Francisco Castillo, David Ramírez, María C. Ramos, Blanca Martinez-Arribas, Elisabeth Domingo-Contreras, Thomas A. Mackenzie, Carlos Peña-Varas, Sven Lindemann, Fernando Montero, Fredderick Annang, Francisca Vicente, Olga Genilloud, Dolores González-Pacanowska, Rosario Fernandez-Godino

**Affiliations:** ^1^ Fundación MEDINA, Granada, Spain; ^2^ Departamento de Farmacología, Facultad de Ciencias Biológicas, Universidad de Concepción, Concepción, Chile; ^3^ Instituto de Parasitología y Biomedicina Lopez-Neyra, Consejo Superior de Investigaciones Científicas, Granada, Spain; ^4^ Doctorado en Biotecnología Molecular, Facultad de Ciencias Biológicas, Universidad de Concepción, Concepción, Chile; ^5^ Strategic Innovation, Merck Healthcare KGaA, Darmstadt, Germany; ^6^ Department of Physical Chemistry and Institute of Biotechnology, Universidad de Granada, Granada, Spain

**Keywords:** COVID-19, betacoronavirus, high-throughput sequencing M-PRO thermal shift, molecular dynamics simulations, molecular docking, antiviral agents

## Abstract

The SARS coronavirus 2 (SARS-CoV-2) epidemic remains globally active. The emergence of new variants of interest and variants of concern (VoCs), which are potentially more vaccine-resistant and less sensitive to existing treatments, is evident due to their high prevalence. The prospective spread of such variants and other coronaviruses with epidemic potential demands preparedness that can be met by developing fast-track workflows to find new candidates that target viral proteins with a clear *in vitro* and *in vivo* phenotype. Mpro (or 3CLpro) is directly involved in the viral replication cycle and the production and function of viral polyproteins, which makes it an ideal target. The biological relevance of Mpro is highly conserved among betacoronaviruses like HCoV-OC43 and SARS-CoV-2, which makes the identification of new chemical scaffolds targeting them a good starting point for designing broad-spectrum antivirals. We report an optimized methodology based on orthogonal cell-free assays to identify small molecules that inhibit the binding pockets of both SARS-CoV-2-Mpro and HCoV-OC43-Mpro; this blockade correlates with antiviral activities in HCoV-OC43 cellular models. By using such a fast-tracking approach against the Open Global Health Library (Merck KGaA), we have found evidence of the antiviral activity of compound OGHL98. *In silico* studies dissecting intermolecular interactions between OGHL98 and both proteases and comprising docking and molecular dynamics simulations (MDSs) concluded that the binding mode was primarily governed by conserved H-bonds with their C-terminal amino acids and that the rational design of OGHL98 has potential against VoCs proteases resistant to current therapeutics.

## 1 Introduction

As coronaviruses continue their global spread, new variants of concern (VoCs) are constantly being detected by genetic surveillance. This has raised a need for the identification of effective therapeutics that overcome the decreased success and increased resistance to existing antivirals (Tan et al., 2022). Mpro mediates viral replication, and it is directly linked to the infection spread in host organisms, which makes it a straightforward target. Rational design studies that led to the validation of the Mpro inhibitor PF-07321332 offer proof of such a target’s druggability. PF-07321332 (nirmatrelvir) is a peptidomimetic drug designed and developed by Pfizer that blocks Mpro viral proteases of beta and alphacoronaviruses, as demonstrated by a low-throughput enzymatic confirmatory assay (Pang et al., 2023). After a couple of years of research, that pan-inhibitory activity demonstrated effective translation well into animal models and human patients when delivered orally (Owen et al., 2021). Therefore, the direct implementation of state-of-the-art cell-free/biochemical assays in high-throughput screening (HTS) format as primary assays could lead to a more rapid discovery of alternative drug precursors, which may be particularly useful against currently emerging Mpro variants resistant to current inhibitors such as Paxlovid (Ip et al., 2023). Finding alternative drug discovery workflows to identify new pharmacophores to block Mpro is a need that has also been flagged by structure–activity studies with phenylbenzisoselenazol-3(2H)-one (ebselen) derivatives, in which Mpro mutations of concern at the so-called *gatekeeper residues* leading to Mpro hyperactivity were studied by Sahoo et al. (2023).

A medium-throughput version of a cell-free FRET enzymatic assay used on a small subset of classic natural products has led to the identification of quercetin as a SARS-CoV-2-Mpro inhibitor, whose mechanism of action relies on the destabilization of the Mpro target according to the thermal shift assay and *in silico* structural biology studies (Abian et al., 2020). Unfortunately, quercetin has shown a marginal *in vivo* effect as an antiviral agent so far, and its mild therapeutic benefits are hard to correlate with Mpro inhibition. Instead, quercetin might inhibit other viral proteins, such as S proteins, or even furin (Di Petrillo et al., 2022). Such mechanistic ambiguity could be overcome with the use of cell-free HTS setups to screen other chemical spaces, such as libraries of synthetic small molecules, to detect inhibitors of higher specificity for the Mpro target. Ideally, this approach should be coupled with secondary assays quickly converging toward bioactive compounds with better broad-spectrum antiviral profiles and absorption, distribution, metabolism, and excretion (ADME) profiles than quercetin.

To test this premise, we have performed a thermal shift assay with the Open Global Health Library (OGHL) (Merck KGaA, Darmstadt, Germany), which is comprised of 250 bioactive synthetic small molecules with demonstrated anti-infective applications (Abraham et al., 2020), but it has never been assayed against coronaviral proteases. The micromolar inhibitory activity of the best compound, OGHL98, a SARS-CoV-2-Mpro destabilizer (ΔT_m_ = −4.5°C ± 0.3°C), was further validated against SARS-CoV-2-Mpro and HCoV-OC43-Mpro (two proteases that share 48.5% of the amino acid sequence identity) in the respective FRET enzymatic assays. To understand how this compound was blocking both Mpro proteases, we designed a computational pipeline including molecular docking followed by long-term molecular dynamics simulations (MDSs). According to such *in silico* structural studies, the broad-spectrum inhibitory activity against these betacoronaviral proteases relies on a conserved network of intermolecular hydrogen bonds established between the C-terminal residues of each protease and the 4-(methylcarbamoyl) benzoic acid moiety of the OGHL98 compound. Finally, detectable antiviral activity was confirmed for OGHL98 against the HCoV-OC43 virus in the micromolar concentration range (cytopathic half maximal effective concentration, EC_50_ value of 33 μM; maximum viral RNA inhibition >50% at 7.5 μM). Future studies are required to confirm the promising ADME/Tox profile predicted in this work and further improve the potency/selectivity of OGHL98 and its benzoic acid moiety using medicinal chemistry tools. More importantly, the orthogonal workflow presented here and based on cost-effective cell-free assays has been demonstrated to be efficient at feeding computational rational design workflows with interesting inhibitors, which delineates a straightforward discovery workflow to be implemented on other cysteine proteases from viruses with pandemic potential.

## 2 Materials and methods

### 2.1 Recombinant production of SARS-CoV-2 and HCoV-OC43 Mpro cysteine proteases

The original pGEX-6p-1 plasmid was donated by Professor Yang’s laboratory. Each plasmid encodes an N-terminal GST tag, followed by the SARS-CoV family autocleavage site (TSAVLQSGFRK) that allows the *in vivo* release of the final C-terminal SARS-CoV-2 and HCoV-OC43 Mpro-His_6x_ used for our protein studies (Xue et al., 2007). The pGEX-6p-1 plasmid (100 ng) was transformed into 20 μL of BL21-RIPL competent cells (Agilent™). Bacterial cultures with successful transformants were grown in 250 mL of LB/ampicillin (100 μg/mL)/chloramphenicol (34 μg/mL) media at 37°C overnight. Then, 4 L of LB/ampicillin (100 μg/mL) were inoculated (1/100 dilution) and incubated at 37°C until OD_600_ 0.8. Overexpression was induced with 1 mM isopropyl-1-thio-β-D-galactopyranoside (IPTG) at 18°C overnight. Cells were harvested by centrifugation at 4°C for 10 min at 7,000 g and re-suspended in lysis buffer (Tris 20 mM, pH 8). Cell lysis was achieved by sonication, from which the debris were removed by centrifugation at 4°C for 30 min at 10,000 g. Mpro-His_6x_ protein was captured from the filtered supernatant using a cobalt HiTrap Column (Cytiva™) and then eluted with 250 mM imidazole. Eluted Mpro-His_6x_ was then buffer-exchanged in lysis buffer for further purification using a HiTrap™ Capto™ Q ImpRes Anion Exchanger (Cytiva™). Fractions of purity above 95% were eluted at 300 mM NaCl, which was buffer-exchanged in phosphate-buffered saline (PBS) before use.

### 2.2 OGHL compound library

The Open Global Health Library, comprising 250 synthetic small molecules, was donated by Merck KGaA™ (Darmstadt, Germany) upon request via https://www.merckgroup.com/en/research/open-innovation/biopharma-open-innovation-portal/open-global-health-library.html. All compounds and controls in this study were provided as 10 mM DMSO stocks and had analytically confirmed purities >90%.

### 2.3 Thermal shift assay

SYPRO Orange (Thermo Fisher Scientific™) was employed as an extrinsic fluorescent probe. An assay master mix containing 5x SYPRO and SARS-CoV-2 Mpro 3 μM in PBS was dispensed into 384-well microplates containing the compound library, which was assayed in 2% DMSO and 100-µM final concentration (Abian et al., 2020). Negative controls contained the master mix and an equivalent volume of DMSO. Positive controls contained the master mix and 200 µM quercetin. Unfolding curves were registered from 20°C to 95°C at a 0.5°C/min scan rate in a Bio-Rad™ CFX 384 qPCR real-time thermal cycler using default HEX filter settings. The midpoint unfolding temperature, T_m_, was calculated in each well as the inflection point and compared to the controls. Primary destabilizing hits were considered using a threshold of T_m_ shift ≤ −2.0°C.

### 2.4 Enzymatic inhibitory assay

Primary hits at 2x concentration and 2% DMSO were pre-incubated with 4 µM SARS-CoV-2-Mpro or HCoV-OC43 enzyme in the assay buffer (20 mM Tris-HCl, pH 7.3, 100 mM NaCl, 1 mM EDTA, and 1 mM TCEP) for 30 min in a low-volume 384-well plate (Qiao et al., 2021). Enzyme activity was monitored on an EnVision Multilabel Plate Reader (PerkinElmer™) at Ex/Em of 320/405 nm after the addition of an equivalent volume of 10 μL of a 2x concentration of the 40 µM peptide substrate MCA-AVLQSGFR-Lys (Dnp)-K. The labeled peptide was purchased from JPT™ as a lyophilized powder (purity >95%). The enzymatic reaction was monitored until reaching equilibrium, according to the end-point inhibitory assay, using quercetin as the positive control and DMSO as the negative control. End-point data for each compound were expressed as fluorescence arbitrary units (*y*-axis) against the log of compound concentration (*x*-axis), from which the IC_50_ values were obtained.

### 2.5 Antiviral activity in a HCoV-OC43 surrogate model

*Reagents and antibodies.* Quercetin, bovine serum albumin, resazurin (Sigma-Aldrich), and ribavirin (Santa Cruz Biotechnology™) were diluted in 100% DMSO. The mouse monoclonal anti-HCoV-OC43 antibody (MAB 2012) was purchased from Millipore™; the Alexa Fluor 488-conjugated anti-mouse secondary antibody and Hoechst 33342 were purchased from Thermo Fisher Scientific™ (Martínez-Arribas et al., 2023).

*Cell culture.* The cell lines used in this study were obtained from the American Type Culture Collection (ATCC). The human lung fibroblast cell line MRC-5 (CCL-171, ATCC) was cultured in minimum essential medium (MEM) (Life Technologies™) supplemented with 10% fetal bovine serum (FBS) (Life Technologies™), 100 units/mL penicillin, and 100 μg/mL streptomycin (Life Technologies™). Cells were incubated at 37°C in a humidified atmosphere of 5% CO_2_ and were periodically analyzed and confirmed to be *mycoplasma* negative.

*Virus production.* The human betacoronavirus HCoV-OC43 (VR-1588, ATCC) was propagated in MRC-5 human cells. In brief, MRC-5 cells were seeded at 90% confluence and inoculated with HCoV-OC43 in infection media (MEM, 2% inactivated FBS, penicillin/streptomycin). Infected cells were incubated for 2 h at 33°C, rocking the flask every 15 min for virus adsorption, and the culture was completed with infection media after adsorption. Infected cells were incubated at 33°C for 5–7 days until more than 50% of the cells presented a cytopathic effect (CPE), resulting in cell death. The infected culture was subjected to three freeze–thaw cycles and centrifuged at 3,000 *g* for 10 min at 4°C to spin down cells and cell debris for virus recovery. Viral particles were recovered from the supernatant, aliquoted in cryotubes, rapidly frozen in a dry-ice/ethanol bath, and stored at −80°C (Martínez-Arribas et al., 2023).

*Batch infection with HCoV-OC43.* MRC-5 cells at 90% confluence were infected with HCoV-OC43 at a multiplicity of infection (MOI) of 0.1. Virus adsorption was performed for 2 h at 33°C, rocking the cells every 15 min, and then the infected cells were incubated for 24 h at 33°C before seeding into 96-well plates (Martínez-Arribas et al., 2023).

*CPE inhibition and cytotoxicity assays.* Infected cells were washed, trypsinized, and seeded in plates containing the compounds at a cellular concentration of 2×10^4^ cells/well in infection media. The plates were incubated at 37°C for 96 h in the presence of the compounds. Infection media were aspirated 5 days after infection, and 120 μL of infection media containing 20% resazurin was added per well. Infected cells treated with 400 µM ribavirin and infected cells with 0.2% DMSO were used as the positive and negative controls, respectively. MRC-5 cells were seeded/well in 96-well plates containing the compounds. After 96 h, the cells were incubated with 20% resazurin for 2 h at 37°C. MRC-5 cells treated with 50 μM of tamoxifen were used as the negative control (100% cell death reference), while positive controls corresponded to MRC-5 cells incubated in the presence of 0.2% DMSO. Fluorescence was determined at 550–590 nm using a Tecan™ Infinite Plate Reader (Martínez-Arribas et al., 2023).

*RNA isolation and RT-PCR*. Viral RNA from the supernatants was purified using the Macherey-Nagel NucleoSpin RNA Kit. RT-qPCR was performed in a single step using the One-Step TB Green PrimeScript RT-PCR Kit II (Takara Bio™). The HCoV-OC43 nucleocapsid gene was amplified with the following primers: the forward primer 5′ AGC​AAC​CAG​GCT​GAT​GTC​AAT​ACC-3′ and the reverse primer 5′ AGC​AGA​CCT​TCC​TGA​GCC​TTC​AAT-3. A standard curve was generated with purified HCoV-OC43 RNA (Vircell™) (Min et al., 2020; Martínez-Arribas et al., 2023).

*Immunofluorescence of HCoV-OC43.* For HCoV-OC43 detection, 4 days after infection, cells were fixed for 20 min with 4% paraformaldehyde and permeabilized for 10 min with 0.4% Triton X-100. After 1 h of blocking with 5% BSA, the cells were incubated O/N with anti-HCoV-OC43. Cells were washed and incubated for 1 h with the Alexa Fluor 488-conjugated anti-mouse secondary antibody (Thermo Fisher Scientific™) and then washed and incubated for 20 min with Hoechst 33342 for nuclei staining. Digital images were captured using the Operetta CLS High Content Analysis System (PerkinElmer™) with a ×5 air objective. The number of nuclei and the number of cells positive for HCoV-OC43 staining were determined, and the percentage of infection was expressed as the ratio of HCoV-OC43 positive cells/total nuclei (Martínez-Arribas et al., 2023).

*Data analysis.* CPE inhibition activities of non-cytotoxic compounds were determined using Equation (1):

$$CPE\;Inhibition\;\left(\%\right)=\frac{\left({Fluo}_{well}-{Fluo}_{neg}\right)}{\left({Fluo}_{pos}-{Fluo}_{neg}\right)}x\;100,$$ (1)

where Fluo_well_ is the measured fluorescence of each well, Fluo_pos_ is the average fluorescence of the positive control (infected MRC-5 cells 0.2% DMSO), and Fluo_neg_ is the average fluorescence of the negative control (non-infected cells).

Cytotoxicity: cellular cytotoxicity was determined using Equation (2):

$$Viability\;\left(\%\right)=\frac{\left({Fluo}_{well}-{Fluo}_{neg}\right)}{\left({Fluo}_{pos}-{Fluo}_{neg}\right)}x\;100,$$ (2)

where Fluo_well_ is the measured fluorescence of each well, Fluo_neg_ is the average fluorescence of the negative control (cells treated with 50 nM tamoxifen), and Fluo_pos_ is the average fluorescence of the positive control (0.2% DMSO) (Martínez-Arribas et al., 2023).

### 2.6 Computational studies

*OC43 MPro modeling:* given that the structure of HCoV-OC43-Mpro has not been solved yet, a homology model was built using the crystal structure of SARS-CoV-2-Mpro as the template (PDB code: 6LU7, (Jin et al., 2020)). Since OC43-MPro is contained in the OC43 replicase polyprotein 1ab (Uniprot ID: P0C6X6), both the sequences (SARS-CoV-2-1ab and OC43-1ab) were aligned. The MPro fraction that showed the optimal sequence alignment was used to build the final model of HCoV-OC43-Mpro, which was optimized using Prime (Schrödinger Suite) and validated using PROCHECK (Jacobson et al., 2004).

*Molecular docking:* new inhibitors, OGHL98 and OGHL43, and control compounds, aspirochlorine and quercetin, were prepared with Maestro and LigPrep. The prepared ligands were docked to the HCoV-OC43-Mpro model and the SARS-CoV-2-Mpro PDB (code: 6LU7) using Glide and Schrödinger Suite (Halgren et al., 2004). Before docking calculations, proteins were prepared using Maestro (Madhavi Sastry et al., 2013), which removed the original ligands, metals, and water molecules. Hydrogens of ionizing residues at pH 7.4 ± 2.0 were then added, and the missing side chains were modeled by Prime. The minimization of the corresponding protein structures was calculated using OPLS3. The same grid box was defined for both targets using the N3 ligand co-crystallized in SARS-CoV2-Mpro as the center of the corresponding boxes. The docking was then performed with the Glide standard precision (SP) function (Friesner et al., 2006). The top 10 poses per docked ligand were selected and subjected to rescoring by calculating the binding free energy (ΔG_bind_) with Prime (Jacobson et al., 2002; 2004), which was calculated in terms of the molecular mechanics-generalized born surface area (MM-GBSA). This computational method combines molecular mechanics energy and implicit solvation models, which enables rescoring and correlation between the experimental activities (IC_50_) and the predicted ΔG_bind_. The corresponding ΔG_bind_ values for each ligand–target complex were calculated, as previously reported (Rojas-Prats et al., 2021).

*Molecular dynamics simulations:* the best post-processed docking solutions between the four inhibitors of interest and both Mpro targets (SARS-CoV-2 and HCoV-OC43) were selected according to their best ΔG_bind_ profiles. Such docking solutions were subjected to MDSs using Desmond software (Bowers et al., 2006) and OPLS3e (Roos et al., 2019). To prepare the systems, the ligand–target complexes were solvated with pre-equilibrated water molecules (SPC) in a periodic boundary condition box. Then, the systems were neutralized by adding Na^+^ or Cl^−^ counter-ions at a final concentration of 0.15 M NaCl to simulate the physiological conditions. Next, each system was relaxed using the default Desmond relaxation protocol and then equilibrated for 25 ns with a spring constant force of 1.0 kcal×mol^-1^×Å^-2^, which was applied to the backbone atoms of the proteins and the ligands. The simulations were performed using the NPT ensemble at constant pressure (1 atm), temperature (310 K), and number of atoms using the isothermal−isobaric ensemble and the Nose−Hoover method, with a relaxation time of 1 ps. The MTK algorithm was applied with a time step of 2 fs. Then, the last frame was taken, and a second non-restricted MD was extended until 3 µs if necessary, for which the same conditions described above were applied. Systems were then analyzed using in-house PyMol and VMD scripts.

*Prediction of the ADME/Tox properties*: we computed the physicochemical descriptors, ADME, pharmacokinetic properties, and drug-like nature of the studied compounds using the SwissADME server (Daina, Michielin, and Zoete, 2017a). In brief, 42 descriptors were predicted, including physicochemical, lipophilicity, water solubility, and pharmacokinetic properties. From these descriptors, SwissADME assessed the compounds’ acceptability based on a bioavailability score (drug-likeness).

## 3 Results

The compound OGHL98 was characterized as a novel inhibitor of coronaviral cysteine proteases using protein thermal shift assays and FRET enzymatic assays. To complement the *in vitro* characterization of OGHL98 beyond these cell-free setups, biological activity tests were performed using a model betacoronavirus, HCoV-OC43. The specific molecular interaction profiles with coronaviral proteases used in the enzymatic assays, HCoV-OC43-Mpro and SARS-CoV-2-Mpro, were studied using a computational pipeline. The pipeline included molecular modeling and docking, followed by binding free energy calculations and long-term molecular dynamics simulations. The latest highlighted the intermolecular contacts established by the main pharmacophoric core of OGHL98. We also predicted a promising ADME/Tox profile for OGHL98 to confirm the efficiency of our drug discovery approach in identifying new and developable antiviral inhibitors.

### 3.1 Identification of novel Mpro destabilizers OGHL43 and OGHL98

Overall, the 250 screened compounds from the Open Global Health Library were synthesized to be structurally diverse. As such, these compounds had shown activity in anti-infective screening campaigns against diverse targets such as amebiasis, AMR, Chagas, visceral leishmaniasis, cryptosporidiosis, human African trypanosomiasis, malaria, schistosomiasis, tuberculosis, or soil-transmitted helminthiasis, but they had never been used in screens against viral proteases.

As described in more detail in Supplementary Figure S1, the collection of 250 diverse compounds presented the following overall physicochemical properties: molecular weight range between 200.0 Da and 700.0 Da, partition coefficient (LogP) between −0.1 and 8.0, number of rotatable bonds between 1 and 15, number of hydrogen bond donors between 0 and 5, number of hydrogen bond acceptors between 2 and 12, and total polar surface area between 0 Å^2^ and 180 Å^2^.

**FIGURE 1:**
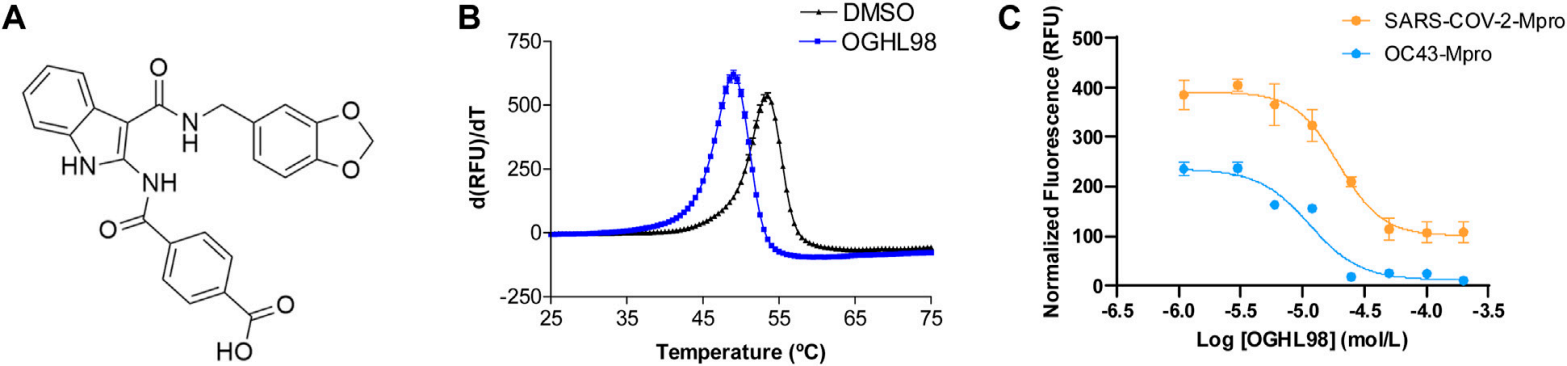
Discovery and characterization of OGHL98 as a novel inhibitor of coronaviral proteases. **(A)** Structure of OGHL98. **(B)** Thermal shift validation reporting a ΔT_m_ = −4.5 ± 0.3 compared to DMSO control. **(C)** FRET enzymatic assay reporting an IC_50_ = 19.6 ± 5.6 µM for SARS-CoV-2-Mpro and 11.4 ± 3.1 µM for HCoV-OC43-Mpro.

Such a library of 250 bioactive compounds and two reference compounds (aspirochlorine and quercetin), previously identified as SARS-CoV-2 antivirals (Singh, Sharma, and Nandi, 2020), were jointly screened against purified SARS-CoV-2-Mpro by thermal shift. The robustness of the screening results was supported by plate quality controls using quercetin (Abian et al., 2020) and DMSO, which yielded a Z′-factor of 0.7 (Lilly et al., 2004). Two hits were selected because they presented a negative Tm shift greater than 2°, which implies a significant destabilization of the protein in the presence of the ligands of interest. Negative shift had already been validated as a feasible mechanism of action to block Mpro with non-covalent small molecules like quercetin (Abian et al., 2020; Mangiavacchi et al., 2021). Then, the two new small molecules, OGHL98 and OGHL43, and the reference compounds aspirochlorine and quercetin were subjected to a functional enzymatic FRET assay with comparable resolution (Z′-factor = 0.6; assay window >5), in which quercetin, as the positive control compound, presented an expected IC_50_ of 28.2 ± 11.4 µM (Abian et al., 2020). Unfortunately, aspirochlorine (ΔT_m_ < -10°C, Supplementary Figure S2B), one of the strongest destabilizers found in the primary screen and whose antiviral activity had been postulated by other authors (Singh, Sharma, and Nandi, 2020), could not be orthogonally confirmed as an Mpro inhibitor for interfering with the signal of the FRET enzymatic assay (Supplementary Figure S3B). More importantly, the FRET assay confirmed the blockade of SARS-CoV-2-Mpro proteolysis by the two new destabilizing molecules, OGHL98 and OGHL43. Furthermore, OGHL98 was defined as the top-performing compound (ΔT_m_ = −4.5 ± 0.3; IC_50_ = 19.6 ± 5.6 µM; Figures 1A–C). OGHL43 (ΔT_m_ = −3.7 ± 0.3; IC_50_ ∼ 80 μM; Supplementary Figures S2A, S3A) was validated as another suitable destabilizer but with lower potency. The dose–response activity of these two new OGHL inhibitors implied a rate of confirmed hits of 0.8% for the whole orthogonal screen, which is in line with the expected efficiency and quality standards of the HTS format for cell-free assays (Lilly et al., 2004). Altogether, these results confirmed OGHL98 as the novel SARS-CoV-2-Mpro destabilizer that demonstrated the highest potency in two cell-free orthogonal assays, reporting a specific impact on protein folding and activity.

Complementarily, we recombinantly expressed and purified the HCoV-OC43-Mpro enzyme to test the inhibitory activity of OGHL98 by a FRET enzymatic assay, in which the compound presented an IC_50_ of 11.4 ± 3.1 µM (Figure 1C, blue trace). This inhibitory activity against HCoV-OC43-Mpro is comparable to the one observed in the SARS-CoV-2 FRET assay (IC_50_ of 19.6 ± 5.6 µM; Figure 1C, orange trace). Jointly, the results pointed toward a broad-spectrum mechanism of action for OGHL98, which was capable of blocking, at low micromolar concentrations, two betacoronaviral proteases with a 48.5% amino acid sequence homology. This mechanism of action would justify potential antiviral activity in biological assays measuring the inhibition of the infection by different betacoronaviruses related to HCoV-OC43 and SARS-CoV-2.

### 3.2 HCoV-OC43 surrogate model confirmed the antiviral activity of OGHL98

The Mpro destabilizers that were identified in the thermal shift assay were further validated *in vitro* using biological assays. This way, we expected to link their already defined mechanism of action to a specific antiviral activity in infected cells.

**FIGURE 2:**
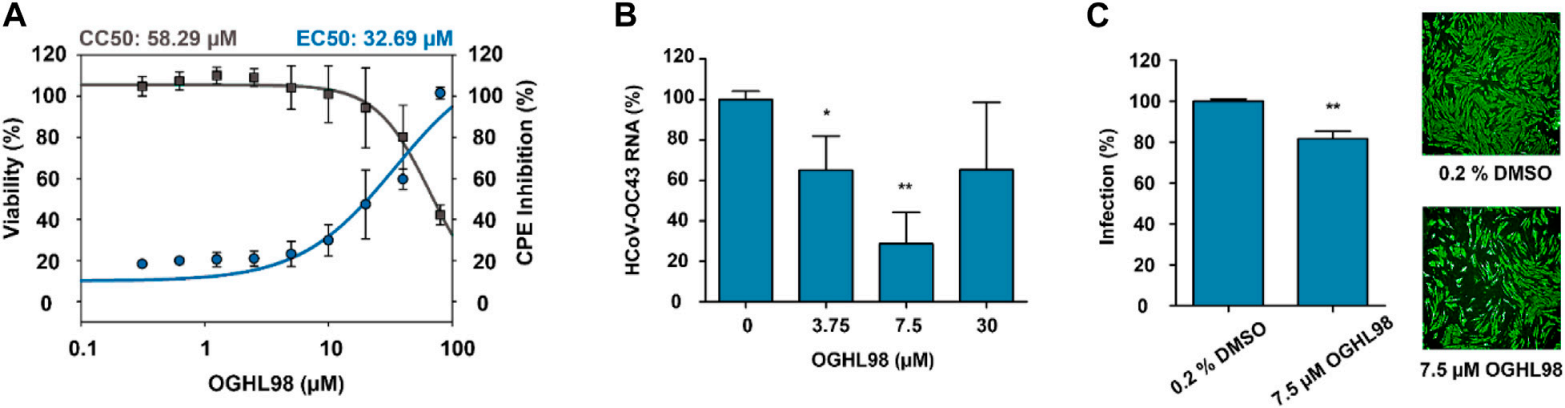
Characterization of the antiviral activity of OGHL98. **(A)** Evaluation of the OGHL98 inhibitor in MRC-5 cells infected with HCoV-OC43 by dose–response curves: CC_50_ (black) and EC_50_ (blue) values for CPE inhibition at 5 days and 96 h post-infection in the presence of the compound. **(B)** HCoV-OC43 RNA levels in infected cells treated with increasing concentrations of OGHL98. **(C)** Representative immunofluorescence images and corresponding bar plots quantifying the percentage of infection of HCoV-OC43 after 72 h in the presence of 7.5 µM OGHL98. Control conditions 0.2% DMSO.

The biological characterization of the best compounds was first addressed through a simple phenotypic assay, in which a successful infection of HCoV-OC43 caused a measurable CPE in the lung cell line MRC-5 (Smee et al., 2017; Martínez-Arribas et al., 2023). CPE inhibition was assessed 96 h after treatment. The EC_50_ values reported for antiviral activity were calculated and compared with the corresponding CC_50_ (half maximal cytotoxic concentration) values, which were obtained in parallel in non-infected MRC-5 cells treated with the same compounds so that non-specific cytotoxic effects could be discriminated. OGHL98 presented the highest activity and selectivity according to an EC_50_ value of 32.69 µM and a CC_50_ value of 58.29 µM (Figure 2A). The EC_50_ value for quercetin was 56.47 µM, and it presented a lower CC_50_ value (28.98 µM, Supplementary Figure S4), thus indicating a lack of selectivity that could explain the ambiguous activities described for this compound (https://pubchem.ncbi.nlm.nih.gov/source/hsdb/3529). In addition, aspirochlorine and OGHL43 were deprioritized because they did not exhibit meaningful CPE inhibition (Supplementary Figure S4).

To link the CPE inhibitory activity of the best compound, OGHL98, to a specific blockade of viral propagation, the RNA levels of HCoV-OC43 were evaluated in the supernatant of infected MRC-5 cells (Min et al., 2020). At a concentration of 7.5 µM, HCoV-OC43 RNA levels were reduced by more than 50% (Figure 2B), orthogonally confirming the inhibitory activity observed in the CPE assay.

Finally, we assessed whether the decrease in viral egress induced by OGHL98 corresponded to reduced replication levels of HCoV-OC43 within host cells. For this, we performed a complementary immunofluorescence study with a monoclonal antibody directed against the nucleocapsid of HCoV-OC43 (Figure 2C). The data confirmed that the infection of MRC-5 cells decreased by 20% after 72 h of treatment with 7.5 µM of OGHL98.

### 3.3 *In silico* studies validated the target engagement of novel destabilizers to SARS-CoV-2-Mpro and HCoV-OC43-Mpro

The identification and confirmation of OGHL98, which blocks viral targets directly involved in the viral replication cycle and the production/function of viral polyproteins like the two coronaviral proteases studied here, could be considered a valid starting point to design broad-spectrum antivirals. Nevertheless, the potency, specificity, ADME, and toxicity profile of OGHL98 must be optimized through an iterative process of rational design that commonly begins with the identification of key pharmacophoric factors for proper ligand binding to the target(s) of interest.

**FIGURE 3:**
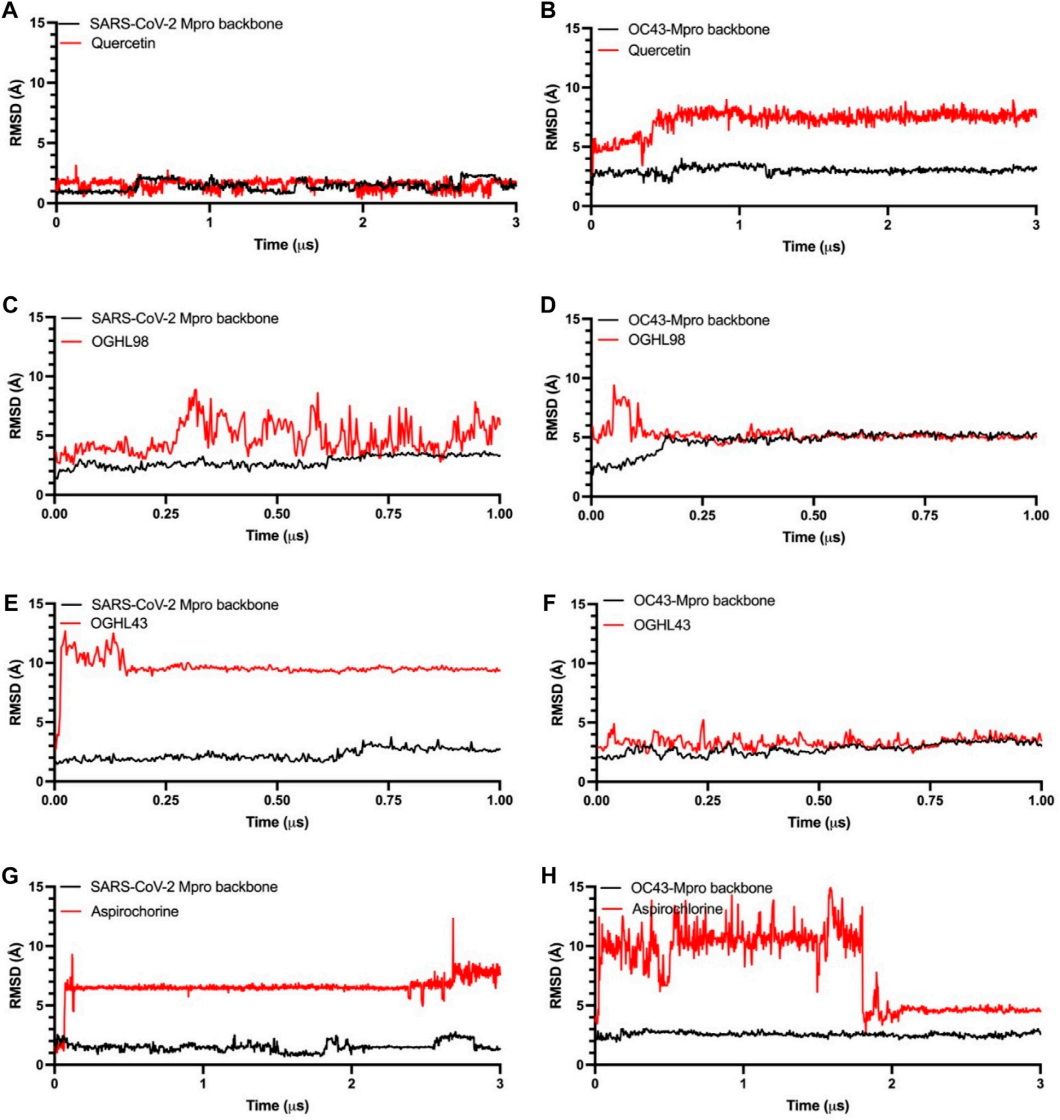
Time dependence of the RMSD for Mpro protein backbones (black) and ligand atoms (red) during the unrestrained molecular dynamics simulations. Reference compound quercetin [panel **(A, B)**], OGHL98 [panel **(C, D)**], OGHL43 [panel **(E, F)**], and aspirochlorine [panel **(G, H)**].

**FIGURE 4:**
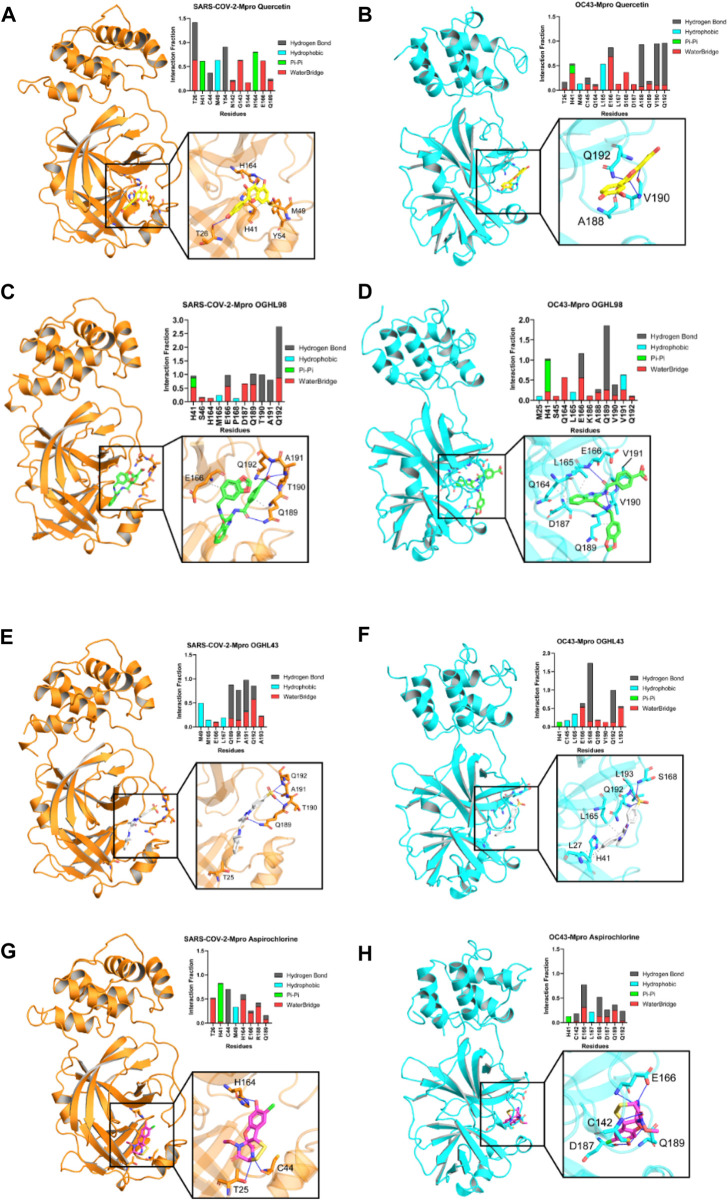
Key intermolecular interactions between Mpro destabilizers and the respective binding sites of Mpro from different betacoronaviruses that were explored by molecular dynamics simulations. Color code is as follows: SARS-CoV-2-Mpro, orange; OC43-Mpro, cyan. Reference compound quercetin [yellow, panel **(A, B)**], OGHL98 [green, panel **(C, D)**], OGHL43 [white, panel **(E, F)**], and aspirochlorine [pink, panel **(G, H)**] are displayed in sticks representation.

To meet the first objective and since the structure of HCoV-OC43-Mpro has not been solved yet, a homology model was built using the crystal structure of SARS-CoV-2-Mpro as the template (PDB code: 6LU7 (Jin et al., 2020), both of which present 48.5% of the amino acid sequence identity. Given that OC43-Mpro is contained within the OC43 replicase polyprotein 1ab (Uniprot ID: P0C6X6), the two sequences (SARS-CoV-2-1ab and OC43-1ab) were aligned, and the Mpro fraction that presented the best sequence alignment was used to build the final HCoV-OC43-Mpro model. Accordingly, further computational studies were performed with the resulting Mpro structures (SARS-CoV-2 and HCoV-OC43) to investigate the molecular determinants of the destabilization induced by the binding of our selected compounds (Figures 3, 4; Supplementary Figure S5).

The newly identified compounds (OGHL43 and OGHL98) were docked into both SARS-CoV-2-Mpro and HCoV-OC43-Mpro targets. Quercetin and aspirochlorine were also docked as control compounds. Then, all docking poses were re-scored according to their predicted binding free energy. Top-scoring solutions were selected to assess the most relevant interactions between each compound and key residues of SARS-CoV-2-Mpro (T26, E166, and Q189), as well as the equivalent residues of HCoV-OC43-MPro (H41, E166, and Q189) (Supplementary Figure S5), which, in all cases, resulted in feasible contacts and geometries and, thus, suggested a specific binding for all cases considered.

The next step was a full characterization of the interactions between each ligand/compound and both targets, SARS-CoV-2-Mpro and HCoV-OC43-MPro, by molecular dynamics simulations. We ran long-term MDSs for both control compounds quercetin and aspirochlorine (3 µs) and the novel inhibitors OGHL43 and OGHL98 (1 µs). Root-mean-square deviation (RMSD) profiles for both protein backbones remained stable and reached equilibrium during the whole set of MD trajectories (Figure 3, black traces). In the RMSD profiles following quercetin’s (Figures 3A, B, red traces) interaction with SARS-CoV-2-Mpro and HCoV-OC43-Mpro, quercetin was observed to interact stably with the binding site during the whole simulation. In the RMSD profiles between the other control compound, aspirochlorine, and SARS-CoV-2-Mpro (Figure 3G), aspirochlorine rotated ∼180° at ∼100 ns and then moved toward a contiguous region of the binding site that was richer in beta-sheet folding. This rearrangement in aspirochlorine translated into RMSD changes of ∼6 Å within the protein, after which aspirochlorine adopted a new conformation that remained stable until the end of the 3 µs simulation. When it comes to the aspirochlorine/HCoV-OC43-MPro trajectory (Figure 3H), the chlorine group of this small molecule altered its initial position when exposed to the solvent, subsequently moved around the perimeter of the binding site of HCoV-OC43-Mpro for the first 1.8 μs of the simulation, and then returned to its initial position, but this time with the chlorine group pointing toward HCoV-OC43-Mpro, which was a conformation that remained stable until the end of the simulation. As described in the RMSD profiling of aspirochlorine, OGHL43 also rotated ∼180° when it interacted with SARS-CoV-2-Mpro at early time points in the trajectory (Figure 3E) and then remained in the binding site of SARS-CoV-2-Mpro during the rest of the simulation, which meant that OGHL43 adopted a binding mode consistent with the one observed in the RMSD interaction profile of the same OGHL43 compound with HCoV-OC43-Mpro (Figure 3F), and it also matched with the corresponding RMSD profiles of OGHL98 (Figures 3C, D). Therefore, from the RMSD profiles of the newly studied ligands, it can be inferred that both OGHL43 and OGHL98 establish stable contacts with both Mpro targets and have common features with the RMSD profiles of the control compounds.

To better understand the intermolecular forces that drive the interactions of each ligand with the binding site of SARS-CoV-2-Mpro, we built interaction profiles where we plotted the corresponding fraction of the trajectory during which each interaction remained stable (Figure 4). For the main reference compound, quercetin, the interaction pattern observed was comparable to that of previous reports that described quercetin as prone to interact with N-terminal β3/αA residues of SARS-CoV-2-Mpro, such as M49 (interaction fractions >0.5, Figure 4A). Our analysis also concurred with the literature in the identification of frequent contacts between quercetin and β10-11 residues of the protein (N142-E166). In addition, the novel compounds OGHL98 (Figure 4C) and OGHL43 (Figure 4E) shared frequent intermolecular hydrogen bonds and water bridges established with two sets of residues, M165-L167 (β11-12) and Q189-A193, located at the C-terminal of SARS-CoV-2-Mpro. In the case of OGHL98, such intermolecular interactions were mostly driven by the 4-(methylcarbamoyl) benzoic acid moiety, which participated in strong water bridges and hydrogen bonds with key residues Q189–Q192 and with neighboring residues from the linker T190–A191 (Figure 4C) (Abian et al., 2020; Cho et al., 2021).

To further assess whether the molecular determinants detected in the MD simulations with our set of inhibitors and SARS-CoV-2-Mpro could justify the enzymatic inhibition that was also observed *in vitro* for HCoV-OC43-Mpro, we built similar binding profiles for this target (45.8% sequence identity), which is considered to be relatively conserved and for which a relatively homologous binding site had been predicted (Berry, Fielding, and Gamieldien, 2015). Notably, intermolecular contacts established between the benzoic moiety of the main inhibitor OGHL98 and the key residues were highly preserved across the whole set of trajectories for the two proteins (Figures 4C, D).

In addition, the rest of the evaluated compounds were found to be prone to establishing favorable hydrogen bonds with a comparable set of C-terminal residues from both HCoV-OC43-Mpro and SARS-CoV-2-Mpro, as shown in the rest of the panels of Figure 4, with reference compound aspirochlorine included (Figures 4G, H). This is important because common intermolecular interaction patterns would justify a common target destabilization mechanism when bound to homologous viral proteases, enabling a match between wet and dry lab results and paving the way for further rational design efforts on OGHL98.

### 3.4 Predictive ADME profiling of OGHL98 using physicochemical descriptors

The SwissADME server was used to predict the ADME, pharmacokinetic properties, and drug-like properties of the best antiviral inhibitor OGHL98, which was compared with the internal reference compounds aspirochlorine and quercetin (Daina, Michielin and Zoete, 2017b) plus the gold-standard inhibitors (Antonopoulou et al., 2022; Sahoo et al., 2023) like covalent inhibitor ebselen or specific inhibitors of Mpro from SARS-CoV-2 like ML-188 (Tables 1, 2).

**TABLE 1:** Physicochemical and pharmacokinetic descriptors of OGHL98 and quercetin calculated with SwissADME.

Compound	Physicochemical properties					Lipophilicity	Water solubility	Pharmacokinetics		
	MW^a^	RB^b^	HB-A^c^	HB-D^d^	TPSA^e^	Consensus log Po/w^f^	Solubility (mol/L)	GI abs^g^	BBB^h^	log Kp (cm/s)^i^
OGHL98	457.43	8	6	4	129.75	3.26	8.92E-08	High	No	−6.34
Quercetin	302.24	1	7	5	131.36	1.23	6.98E-04	High	No	−7.05
Ebselen (IC_50_ = 0.67 uM)	274.18	1	1	0	22.00	1.75	1-20E-04	High	Yes	−6.00
Aspirochlorine	360.79	1	5	2	138.7	0.78	1.42e-03	High	No	−7.78
Perampanel (IC_50_ = 100–250 uM)	349.38	3	3	0	58.68	3.71	2.33e-05	High	Yes	−5.99
F01 (IC_50_ = 54 uM)	286.71	3	3	1	59.06	2.34	6.51e-04	High	Yes	−6.56
ML188 (IC_50_ = 2.5 uM)	431.51	9	4	1	108.36	3.27	4.38e-05	High	No	−6.72
ML300 (IC_50_ = 4.99 uM)	433.54	9	4	1	75.44	4.06	3.48e-06	High	No	−5.42

^a^ Molecular weight (g/mol).

^b^ Number of rotatable bonds.

^c^ Number of hydrogen bond acceptors.

^d^ Number of hydrogen bond donors.

^e^ Topological polar surface area (Ertl, Rohde, and Selzer, 2000).

^f^ Average of iLOGP, XLOGP, WLOGP, MLOGP, and SILICOS-IT predictions (Daina, Michielin, and Zoete, 2017b).

^g^ Gastrointestinal absorption.

^h^ Blood–brain barrier permeation.

^i^ Skin permeation: QSPR model (Potts and Guy, 1992).

**TABLE 2:** Drug-likeness properties of OGHL98 and quercetin calculated using SwissADME.

Compound	Lipinski # violations^a^	Ghose # violations^b^	Veber # violations^c^	Egan # violations^d^	Muegge # violations ^e^
OGHL98	0	0	0	0	0
Quercetin	0	0	0	0	0
Ebselen (IC_50_ = 0.67 uM)	0	0	0	0	0
Aspirochlorine	0	0	0	1	0
Perampanel (IC_50_ = 100–250 uM)	0	0	0	0	0
F01 (IC_50_ = 54 uM)	0	0	0	0	0
ML188 (IC_50_ = 2.5 uM)	0	0	0	0	0
ML300 (IC_50_ = 4.99 uM)	0	0	0	0	0

^a^ Lipinski (Pfizer) filter (Lipinski et al., 2001): MW ≤ 500; MLOGP ≤4.15; N or O ≤ 10; NH or OH ≤ 5.

^b^ Ghose filter (Ghose, Viswanadhan and Wendoloski, 1999): 160 ≤ MW ≤ 480; −0.4 ≤ WLOGP ≤5.6; 40 ≤ MR ≤ 130; 20 ≤ atoms ≤70.

^c^ Veber (GSK) filter (Veber et al., 2002): rotatable bonds ≤10; TPSA ≤140.

^d^ Egan (Pharmacia) filter (Veber et al., 2002): WLOGP ≤5.88; TPSA ≤131.6.

^e^ Muegge (Bayer) filter (Muegge, Heald, and Brittelli, 2001): 200 ≤ MW ≤ 600; −2 ≤ XLOGP ≤5; TPSA ≤150; number of rings ≤7; number of carbon atoms >4; number of heteroatoms >1; number of rotatable bonds ≤15.

Importantly, our best inhibitor OGHL98 had a molecular weight (MW) < 500 g/mol, which was within the optimal range for a potential lead drug. All the remaining physicochemical descriptors, like the number of rotatable bonds, hydrogen bond acceptors (HB-A), donors (HB-D), topological polar surface area (TPSA), lipophilicity index, and water solubility, were also in the corresponding acceptable ranges (Table 1, left). A similar trend was observed for the pharmacokinetic properties (Table 1, right). The absence of drug-likeness violations in the complementary analysis, which is summarized in Table 2, further confirmed the effectiveness of our drug-discovery workflow in identifying feasible and developable small-molecule inhibitors.

## 4 Discussion

During the SARS-CoV-2 pandemic, the testing of a small subset of classic natural products led to the identification of quercetin as a SARS-CoV-2-Mpro inhibitor, whose mechanism of action relies on the non-covalent binding and destabilization of this macromolecule, and it was used as a starting point to design quercetin derivatives having a scope as antivirals (Abian et al., 2020; Mangiavacchi et al., 2021). Unfortunately, quercetin has shown a marginal *in vivo* effect as an antiviral agent (Di Petrillo et al., 2022), and its mild therapeutic benefits are hard to correlate with Mpro inhibition. This fact, together with the potential spread of new variants of interest and concern, which are likely to be more vaccine-resistant and less sensitive to existing Mpro inhibitors in the clinic (Ip et al., 2023; Sahoo et al., 2023), raised the question of how feasible it would be to implement cell-free HTS setups to explore alternative chemical spaces like synthetic small molecules and quickly detect specific destabilizers of the Mpro target with better antiviral profiles than quercetin. Ideally, the characterization of these new destabilizers will provide medicinal chemists with new chemical scaffolds so that they can be optimized into broad-spectrum antivirals, which are considered ideal preparedness tools against future pandemics.

A good starting point to meet these needs is the successful implementation of two orthogonal thermal-shift and FRET assays in the HTS format, which have led to the discovery of a novel small molecule called OGHL98 by screening the Open Global Health Library (Merck KGaA). To date, OGHL98 has been described as a mere PDE5 inhibitor with the potential to treat erectile dysfunction and pulmonary arterial hypertension (Ahmed, Geethakumari, and Biswas, 2021). In this work, we have characterized a new activity for OGHL98 by confirming its potential as a developable antiviral against the infection of HCoV-OC43, which is a biosafety level-2 coronavirus. The mechanism of action of OGHL98 involves the enzymatic inhibition of two conserved viral proteases, SARS-CoV-2-Mpro and HCoV-OC43-Mpro, as demonstrated by the cell-free setups. Therefore, this fast-tracking methodology can lead to the development of novel and efficient broad-spectrum antivirals.

It is important to note that the design of OGHL98 analogs that increase EC_50_/CC_50_ selectivity is required to meet general pre-clinical standards (Sun et al., 2022). Furthermore, medicinal chemistry efforts will also be required to increase the potency that OGHL98 has shown *in vitro*. In this line, the long-term MD simulations suggest *in silico* optimization of the hydrogen bonds between the nitrogen atoms of OGHL98 (amide groups and indole moiety) and the backbone of the E166 residue in the wild-type Mpro. These contacts might be further improved to efficiently block VoC mutations within this region of Mpro, which is responsible for the resistance against nirmatrelvir or ensitrelvir (Ip et al., 2023). It is important to highlight that more than 50% of FDA-approved drugs are nitrogen-containing molecules, the majority of which are N-heterocyclic small molecules (Kerru et al., 2020), like OGHL98, which supports the proposed strategy. Alternatively, it might be possible to design OGHL98 analogs with strengthened intermolecular interactions between the carboxylic acid group of the parent compound and the Q189 residue in wild-type Mpro. This second strategy could also be extended to another set of interesting Mpro VoC mutants, such as Q189K, ΔQ189, Q189H, Q189L, Q189P, Q189G, and Q189S (Najjar-Debbiny et al., 2023).

Finally, it is important to note that OGHL98 reported here fulfills all the drug-likeness properties (similar to control compounds) to be a potential drug (Tables 1, 2) according to the predicted physicochemical descriptors and pharmacokinetic properties (Daina, Michielin, and Zoete, 2017a). The computational pipeline and the experimental design advocate for the oral use of OGHL98 analogs of optimized potency, selectivity, and ADME/Tox profile.

## 5 Conclusion

We have demonstrated that the antiviral activity of OGHL98 exceeds the biological performance of the previously reported Mpro destabilizer, quercetin (Abian et al., 2020). This work reveals that OGHL98 acts as an inhibitor of two (SARS-CoV-2 and OC43) coronaviral proteases, which supports the theory that broad-spectrum inhibition against betacoronaviral proteases could be achieved using rational design approaches.

More importantly, the cell-free primary assays combined here to find hits define a cost-effective early drug-discovery workflow amenable for the screening of massive small-molecule libraries to feed the computational rational design campaigns and further validate more potent pharmacophores with double-digit selectivity indexes.

Such a future line of research is of special interest if implemented against hyperactive Mpro variants resistant to current inhibitors or other viral proteases from viruses with pandemic potential, for which no specific inhibitors have been identified so far.

## 6 Scope statement

The emergence of new variants of concern of coronaviruses, potentially more vaccine-resistant and less sensitive to existing treatments, is evident due to their high prevalence. A prospective spread of such VoCs demands a preparedness that can be met by fast-tracking workflows aiming at viral protein targets with a clear *in vitro*/*in vivo* phenotype. Mpro (or 3CLpro) is directly involved in the viral replication cycle and the production and function of viral polyproteins. These roles are conserved among betacoronaviruses like HCoV-OC43 and SARS-CoV-2, which makes the identification of new inhibitors for them a good starting point for designing broad-spectrum antivirals. We report an optimized methodology based on orthogonal cell-free assays to identify small molecules that inhibit the binding pockets of both SARS-CoV-2-Mpro and HCoV-OC43-Mpro, whose blockade correlates with antiviral activities in HCoV-OC43 cellular models. By using such a fast-tracking approach against the Open Global Health Library (Merck KGaA), we have found evidence of new antiviral activity for compound OGHL98. *In silico* molecular dynamics dissecting intermolecular interactions between OGHL98 and both proteases concluded that the binding mode was primarily governed by conserved H-bonds with their C-terminal amino acids and that the rational design of OGHL98 has potential against VoC proteases.

## Data Availability

The original contributions presented in the study are included in the article/Supplementary Materials, further inquiries can be directed to the corresponding authors.

## References

[B1] Abian O. Ortega-Alarcon D. Jimenez-Alesanco A. Ceballos-Laita L. Vega S. Reyburn H. T. (2020). Structural stability of SARS-CoV-2 3CLpro and identification of quercetin as an inhibitor by experimental screening. Int. J. Biol. Macromol. 164, 1693–1703. 10.1016/j.ijbiomac.2020.07.235 32745548 PMC7395220

[B2] Abraham M. Gagaring K. Martino M. L. Vanaerschot M. Plouffe D. M. Calla J. (2020). Probing the open global Health chemical diversity library for multistage-active starting points for next-generation antimalarials. ACS Infect. Dis. 6 (4), 613–628. 10.1021/acsinfecdis.9b00482 32078764 PMC7155171

[B3] Antonopoulou I. Sapountzaki E. Rova U. Christakopoulos P. (2022). Inhibition of the main protease of SARS-CoV-2 (Mpro) by repurposing/designing drug-like substances and utilizing nature’s toolbox of bioactive compounds. Comput. Struct. Biotechnol. J. 20, 1306–1344. 10.1016/j.csbj.2022.03.009 35308802 PMC8920478

[B4] Berry M. Fielding B. Gamieldien J. (2015). Human coronavirus OC43 3CL protease and the potential of ML188 as a broad-spectrum lead compound: homology modelling and molecular dynamic studies. BMC Struct. Biol. 15 (1), 8. 10.1186/s12900-015-0035-3 25928480 PMC4411765

[B5] Bowers K. J. (2006). “Molecular dynamics---Scalable algorithms for molecular dynamics simulations on commodity clusters,” in Proceedings of the 2006 ACM/IEEE conference on supercomputing - SC ’06 (New York, New York, USA: ACM Press), 84. 10.1145/1188455.1188544

[B6] Cho E. Rosa M. Anjum R. Mehmood S. Soban M. Mujtaba M. (2021). Dynamic profiling of β-coronavirus 3CL M ^pro^ protease ligand-binding sites. J. Chem. Inf. Model. 61 (6), 3058–3073. 10.1021/acs.jcim.1c00449 34124899

[B7] Daina A. Michielin O. Zoete V. (2017a). SwissADME: a free web tool to evaluate pharmacokinetics, drug-likeness and medicinal chemistry friendliness of small molecules. Sci. Rep. 7 (1), 42717. 10.1038/srep42717 28256516 PMC5335600

[B8] Daina A. Michielin O. Zoete V. (2017b). SwissADME: a free web tool to evaluate pharmacokinetics, drug-likeness and medicinal chemistry friendliness of small molecules. Sci. Rep. 7 (1), 42717. 10.1038/srep42717 28256516 PMC5335600

[B9] Di Petrillo A. Orrù G. Fais A. Fantini M. C. (2022). Quercetin and its derivates as antiviral potentials: a comprehensive review. Phytotherapy Res. 36 (1), 266–278. 10.1002/ptr.7309 PMC8662201 34709675

[B10] Ertl P. Rohde B. Selzer P. (2000). Fast calculation of molecular polar surface area as a sum of fragment-based contributions and its application to the prediction of drug transport properties. J. Med. Chem. 43 (20), 3714–3717. 10.1021/jm000942e 11020286

[B11] Friesner R. A. Murphy R. B. Repasky M. P. Frye L. L. Greenwood J. R. Halgren T. A. (2006). Extra precision Glide: docking and scoring incorporating a model of hydrophobic enclosure for Protein−Ligand complexes. J. Med. Chem. 49 (21), 6177–6196. 10.1021/jm051256o 17034125

[B12] Ghose A. K. Viswanadhan V. N. Wendoloski J. J. (1999). A knowledge-based approach in designing combinatorial or medicinal chemistry libraries for drug discovery. 1. A qualitative and quantitative characterization of known drug databases. J. Comb. Chem. 1 (1), 55–68. 10.1021/cc9800071 10746014

[B13] Halgren T. A. Murphy R. B. Friesner R. A. Beard H. S. Frye L. L. Pollard W. T. (2004). Glide: a new approach for rapid, accurate docking and scoring. 2. Enrichment factors in database screening. J. Med. Chem. 47 (7), 1750–1759. 10.1021/jm030644s 15027866

[B14] Ip J. D. Wing-Ho Chu A. Chan W. M. Cheuk-Ying Leung R. Umer Abdullah S. M. Sun Y. (2023). Global prevalence of SARS-CoV-2 3CL protease mutations associated with nirmatrelvir or ensitrelvir resistance. eBioMedicine 91, 104559. 10.1016/j.ebiom.2023.104559 37060743 PMC10101811

[B15] Jacobson M. P. Friesner R. A. Xiang Z. Honig B. (2002). On the role of the crystal environment in determining protein side-chain conformations. J. Mol. Biol. 320 (3), 597–608. 10.1016/S0022-2836(02)00470-9 12096912

[B16] Jacobson M. P. Pincus D. L. Rapp C. S. Day T. J. F. Honig B. Shaw D. E. (2004). A hierarchical approach to all-atom protein loop prediction. Proteins Struct. Funct. Bioinforma. 55 (2), 351–367. 10.1002/prot.10613 15048827

[B17] Jin Z. Du X. Xu Y. Deng Y. Liu M. Zhao Y. (2020). Structure of Mpro from SARS-CoV-2 and discovery of its inhibitors. Nature 582 (7811), 289–293. 10.1038/s41586-020-2223-y 32272481

[B18] Kerru N. Gummidi L. Maddila S. Gangu K. K. Jonnalagadda S. B. (2020). A review on recent advances in nitrogen-containing molecules and their biological applications. Molecules 25 (8), 1909. 10.3390/molecules25081909 32326131 PMC7221918

[B19] Lilly E. Coussens N. P. Sittampalam G. S. Guha R. Brimacombe K. Grossman A. (2004). Assay guidance manual. Available at: https://ncats.nih.gov/expertise/preclinical/agm. 22553861

[B20] Lipinski C. A. Lombardo F. Dominy B. W. Feeney P. J. (2001). Experimental and computational approaches to estimate solubility and permeability in drug discovery and development settings 1PII of original article: S0169-409X(96)00423-1. Adv. Drug Deliv. Rev. 46 (1–3), 3–26. The article was originally published in Advanced Drug Delivery Reviews 23 (1997) 3–25. 1’. 10.1016/S0169-409X(00)00129-0 11259830

[B21] Madhavi Sastry G. Adzhigirey M. Day T. Annabhimoju R. Sherman W. (2013). Protein and ligand preparation: parameters, protocols, and influence on virtual screening enrichments. J. Computer-Aided Mol. Des. 27 (3), 221–234. 10.1007/s10822-013-9644-8 23579614

[B22] Mangiavacchi F. Botwina P. Menichetti E. Bagnoli L. Rosati O. Marini F. (2021). Seleno-functionalization of quercetin improves the non-covalent inhibition of Mpro and its antiviral activity in cells against SARS-CoV-2. Int. J. Mol. Sci. 22 (13), 7048. 10.3390/ijms22137048 34208928 PMC8268238

[B23] Martínez-Arribas B. Annang F. Díaz-González R. Pérez-Moreno G. Martín J. Mackenzie T. A. (2023). Establishment of a screening platform based on human coronavirus OC43 for the identification of microbial natural products with antiviral activity. Microbiol. Spectr. 12, e0167923. 10.1128/spectrum.01679-23 38009959 PMC10783114

[B24] Min J. S. Kim D. E. Jin Y. H. Kwon S. (2020). Kurarinone inhibits HCoV-OC43 infection by impairing the virus-induced autophagic flux in MRC-5 human lung cells. J. Clin. Med. 9 (7), 2230. 10.3390/jcm9072230 32674356 PMC7408680

[B25] Muegge I. Heald S. L. Brittelli D. (2001). Simple selection criteria for drug-like chemical matter. J. Med. Chem. 44 (12), 1841–1846. 10.1021/jm015507e 11384230

[B26] Najjar-Debbiny R. Gronich N. Weber G. Khoury J. Amar M. Stein N. (2023). Effectiveness of Paxlovid in reducing severe coronavirus disease 2019 and mortality in high-risk patients. Clin. Infect. Dis. 76 (3), e342–e349. 10.1093/cid/ciac443 35653428 PMC9214014

[B27] Owen D. R. Allerton C. M. N. Anderson A. S. Aschenbrenner L. Avery M. Berritt S. (2021). An oral SARS-CoV-2 M ^pro^ inhibitor clinical candidate for the treatment of COVID-19. Science 374 (6575), 1586–1593. 10.1126/science.abl4784 34726479

[B28] Pang X. Xu W. Liu Y. Li H. Chen L. (2023). The research progress of SARS-CoV-2 main protease inhibitors from 2020 to 2022. Eur. J. Med. Chem. 257, 115491. 10.1016/j.ejmech.2023.115491 37244162 PMC10201905

[B29] Potts R. O. Guy R. H. (1992). Predicting skin permeability. Pharm. Res. 09 (5), 663–669. 10.1023/A:1015810312465 1608900

[B30] Qiao Z. Wei N. Jin L. Zhang H. Luo J. Zhang Y. (2021). The Mpro structure-based modifications of ebselen derivatives for improved antiviral activity against SARS-CoV-2 virus. Bioorg. Chem. 117, 105455. 10.1016/j.bioorg.2021.105455 34740055 PMC8556866

[B31] Rojas-Prats E. Martinez-Gonzalez L. Gonzalo-Consuegra C. Liachko N. F. Perez C. Ramírez D. (2021). Targeting nuclear protein TDP-43 by cell division cycle kinase 7 inhibitors: a new therapeutic approach for amyotrophic lateral sclerosis. Eur. J. Med. Chem. 210, 112968. 10.1016/j.ejmech.2020.112968 33139113

[B32] Roos K. Wu C. Damm W. Reboul M. Stevenson J. M. Lu C. (2019). OPLS3e: Extending Force Field Coverage for Drug-Like Small Molecules. J. Chem. Theory Comput. 15 (3), 1863–1874. 10.1021/acs.jctc.8b01026 30768902

[B33] Sahoo P. Lenka D. R. Batabyal M. Pain P. K. Kumar S. Manna D. (2023). Detailed insights into the inhibitory mechanism of new ebselen derivatives against main protease (M ^pro^) of severe acute respiratory syndrome coronavirus-2 (SARS-CoV-2). ACS Pharmacol. Transl. Sci. 6 (1), 171–180. 10.1021/acsptsci.2c00203 36650888 PMC9797022

[B34] Singh P. Sharma A. Nandi S. P. (2020). ‘Identification of potent inhibitors of COVID-19 main protease enzyme by molecular docking study’. Available at: https://chemrxiv.org/engage/chemrxiv/article-details/60c74a4e4c89195569ad31e4.

[B35] Smee D. F. Hurst B. L. Evans W. J. Clyde N. Wright S. Peterson C. (2017). Evaluation of cell viability dyes in antiviral assays with RNA viruses that exhibit different cytopathogenic properties. J. Virological Methods 246, 51–57. 10.1016/j.jviromet.2017.03.012 PMC5479350 28359770

[B36] Sun D. Gao W. Hu H. Zhou S. (2022). Why 90% of clinical drug development fails and how to improve it? Acta Pharm. Sin. B 12 (7), 3049–3062. 10.1016/j.apsb.2022.02.002 35865092 PMC9293739

[B37] Tan C. Y. Chiew C. J. Lee V. J. Ong B. Lye D. C. Tan K. B. (2022). Comparative effectiveness of 3 or 4 doses of mRNA and inactivated whole-virus vaccines against COVID-19 infection, hospitalization and severe outcomes among elderly in Singapore. Lancet Regional Health - West. Pac. 29, 100654. 10.1016/j.lanwpc.2022.100654 PMC9716291 36471699

[B38] Veber D. F. Johnson S. R. Cheng H. Y. Smith B. R. Ward K. W. Kopple K. D. (2002). Molecular properties that influence the oral bioavailability of drug candidates. J. Med. Chem. 45 (12), 2615–2623. 10.1021/jm020017n 12036371

[B39] Xue X. Yang H. Shen W. Zhao Q. Li J. Yang K. (2007). Production of authentic SARS-CoV Mpro with enhanced activity: application as a novel tag-cleavage endopeptidase for protein overproduction. J. Mol. Biol. 366 (3), 965–975. 10.1016/j.jmb.2006.11.073 17189639 PMC7094453

