# Supplementary material for: Repurposing the Open Global Health Library for the discovery of novel Mpro destabilizers with scope as broad-spectrum antivirals
Source: Front Pharmacol. 2024 Jul 10;15:1390705. doi: 10.3389/fphar.2024.1390705 (PMC11267763; doi:10.3389/fphar.2024.1390705)
Supplement: Supplementary file 1 [file DataSheet1.docx]

SUPPLEMENTARY MATERIALS


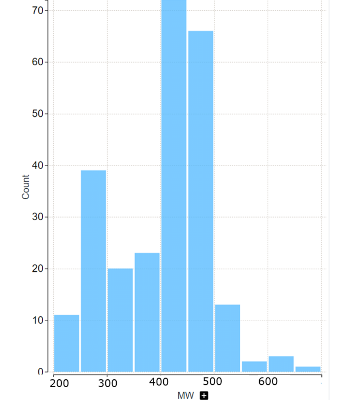
            
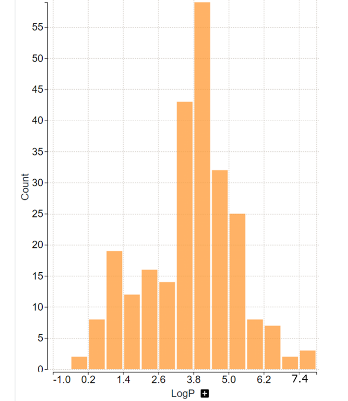
         
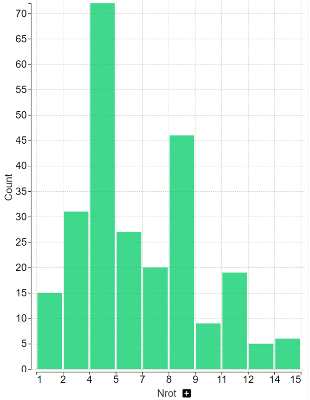


A                                            B                                            C


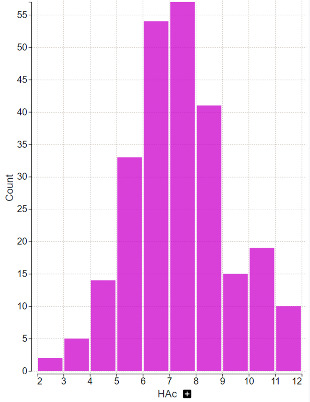
            
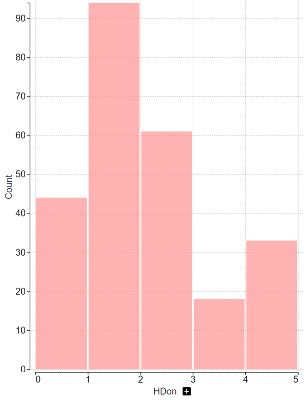
              
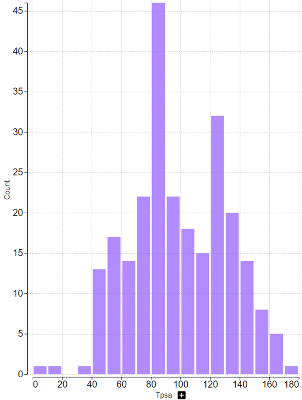


D                                             E                                            F

***Figure S1.*** *Physicochemical properties and descriptors of the 250 diverse compounds from the Open Global Health Library. A) molecular weight (MW) range between 200.0 Da and 700.0 Da; B) partition coefficient (LogP) between -0.1 and 8.0, C) number of rotable bonds (Nrot) between 1 and 15, D) number of hydrogen bond donors of between 0 and 5,  E) hydrogen bond acceptors (Hac) between 2 and 12; and F) total polar surface area (TPSA) between 0 Å^2^ and 180 Å^2^.*

A B C

***Figure S2.*** *Confirmed Mpro destabilizers identified in the thermal shift screening. A) Compound OGHL43 induces a thermal shift or ΔTm of -3.7 ± 0.3 at 100 µM. C) Aspirochlorine induces a thermal shift or ΔTm of -16.3 ± 0.3 at 100 µM. D) Quercetin positive control induces a thermal shift or ΔTm of -6.5 ± 0.5 at 200 µM. All Tm shifts ΔTm are expressed with respect to DMSO reference (2% final).*

A B
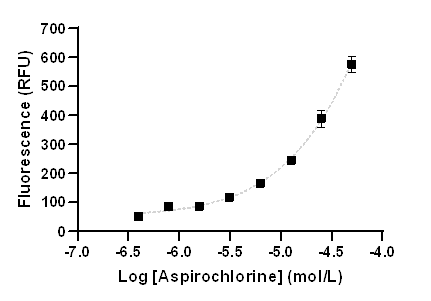
 C
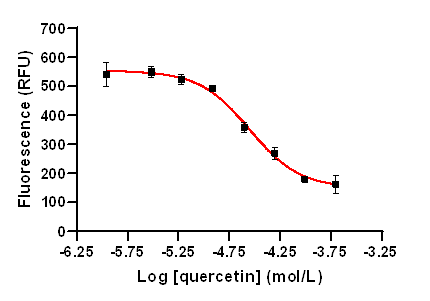


***Figure S3.*** *FRET enzymatic assay reporting the blockade activity of Mpro destabilizers from the thermal shift screening. A) Compound OGHL43 has an IC_50_ of ~ 80 µM. B) Aspirochlorine IC_50_ could not be measured due to presenting a dose-response interference with the fluorescence reporter. C) Quercetin positive control has an IC_50_ of 28.2 ± 11.4 µM.*


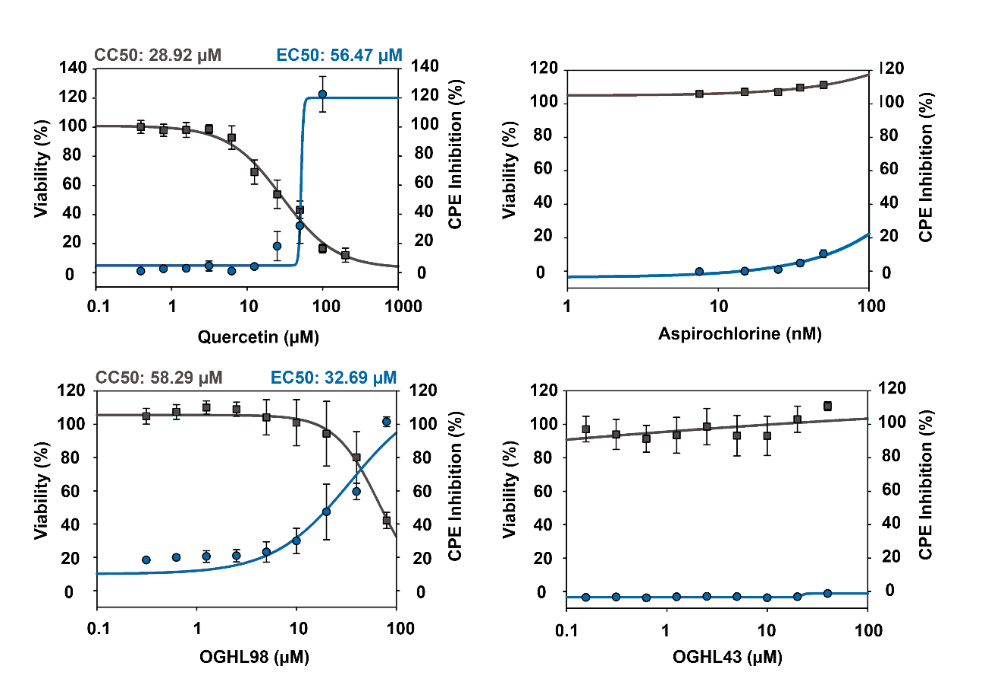


***Figure S4.*** *CPE assay reporting for antiviral activity of the four compounds of interest in our HCoV-OC43 model. Black line corresponds to the best fitting to obtain CC_50_ toxicity values from experimental values shown as black squares. Blue line corresponds to the best fitting to obtain EC_50_ activity values from experimental values shown as blue circles.*


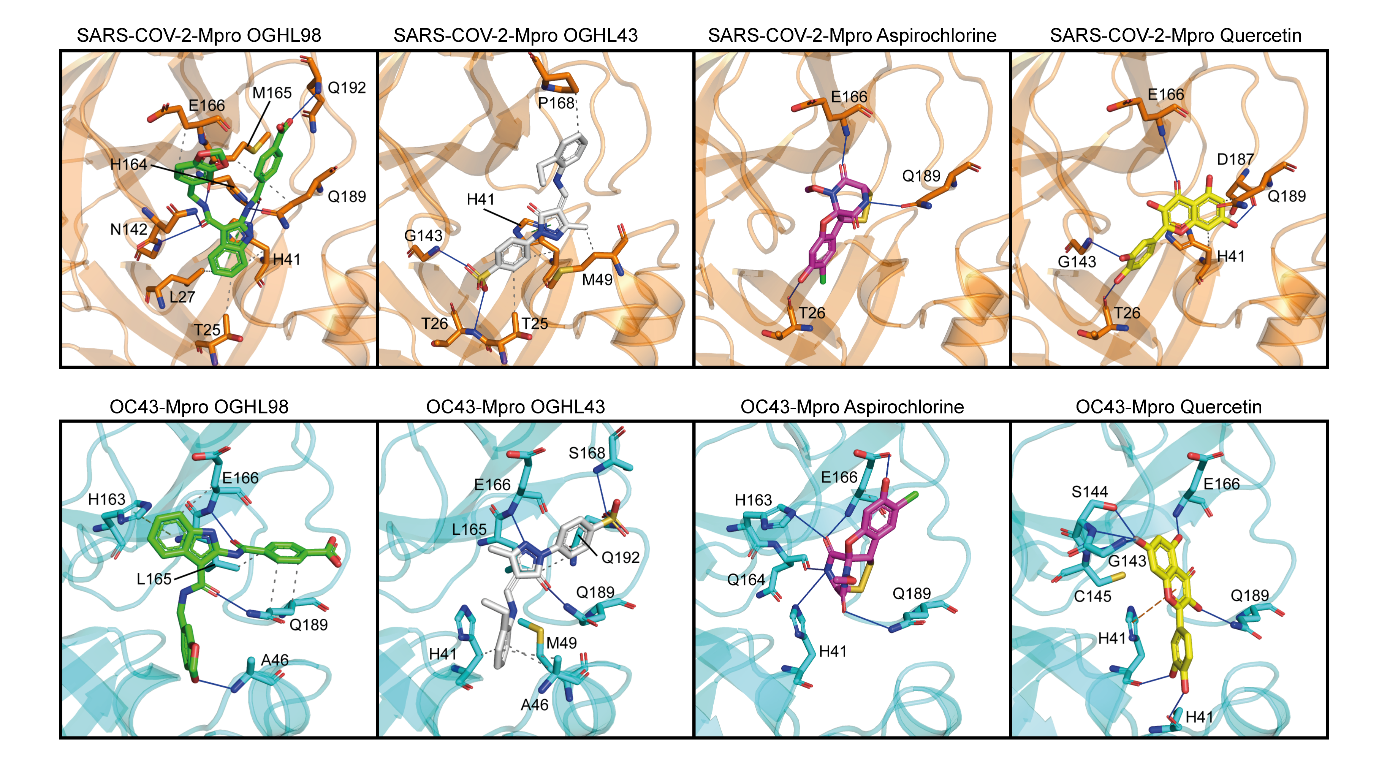


***Figure S5.*** *In silico studies reporting for the molecular determinants driving the intermolecular interactions of the Mpro destabilizers with E166, Q189 and different subsets of residues from the target of interest. Colour code goes as follows. SARS-CoV-2-Mpro: orange (top panels). OC43-Mpro* [21]*: cyan (bottom panels). Compounds OGHL98 (green), OGHL43 (white), aspirochlorine (pink), and quercetin (yellow) are displayed in sticks representation.*
